# Plant Natural Product Formononetin Protects Rat Cardiomyocyte H9c2 Cells against Oxygen Glucose Deprivation and Reoxygenation via Inhibiting ROS Formation and Promoting GSK-3*β* Phosphorylation

**DOI:** 10.1155/2016/2060874

**Published:** 2016-01-06

**Authors:** Yuanyuan Cheng, Zhengyuan Xia, Yifan Han, Jianhui Rong

**Affiliations:** ^1^School of Chinese Medicine, Li Ka Shing Faculty of Medicine, The University of Hong Kong, 10 Sassoon Road, Pokfulam, Hong Kong; ^2^Department of Anesthesiology, Li Ka Shing Faculty of Medicine, The University of Hong Kong, 10 Sassoon Road, Pokfulam, Hong Kong; ^3^Department of Applied Biology and Chemical Technology, Hong Kong Polytechnic University, Hung Hom, Hong Kong

## Abstract

The opening of mitochondrial permeability transition pore (mPTP) is a major cause of cell death in ischemia reperfusion injury. Based on our pilot experiments, plant natural product formononetin enhanced the survival of rat cardiomyocyte H9c2 cells during oxygen glucose deprivation (OGD) and reoxygenation. For mechanistic studies, we focused on two major cellular factors, namely, reactive oxygen species (ROS) and glycogen synthase kinase 3*β* (GSK-3*β*), in the regulation of mPTP opening. We found that formononetin suppressed the formation of ROS and superoxide in a concentration-dependent manner. Formononetin also rescued OGD/reoxygenation-induced loss of mitochondrial membrane integrity. Further studies suggested that formononetin induced Akt activation and GSK-3*β* (Ser9) phosphorylation, thereby reducing GSK-3*β* activity towards mPTP opening. PI3K and PKC inhibitors abolished the effects of formononetin on mPTP opening and GSK-3*β* phosphorylation. Immunoprecipitation experiments further revealed that formononetin increased the binding of phosphor-GSK-3*β* to adenine nucleotide translocase (ANT) while it disrupted the complex of ANT with cyclophilin D. Moreover, immunofluorescence revealed that phospho-GSK-3*β* (Ser9) was mainly deposited in the space between mitochondria and cell nucleus. Collectively, these results indicated that formononetin protected cardiomyocytes from OGD/reoxygenation injury via inhibiting ROS formation and promoting GSK-3*β* phosphorylation.

## 1. Introduction

Acute myocardial infarction (AMI) is a leading cause of human death worldwide [1]. Timely reperfusion effectively reduces short-term mortality in early-reperfusion phase [2]. One of the on-going issues is that reperfusion itself leads to additional injury, causing all forms of cell death and contractile dysfunction of surviving cells [3]. The mitochondrial permeability transition pore (mPTP) is implicated in the pathogenesis of myocardial ischemia-reperfusion injury [4]. Ischemia induces the close of mPTP, whereas reperfusion promotes the mPTP opening [5]. Thus, control of mPTP opening at the early reperfusion is important to protect the heart against reperfusion injury [6]. Among various regulators of mPTP opening, glycogen synthase kinase 3*β* (GSK-3*β*) is a constitutively active serine or threonine protein kinase [7]. GSK-3*β* stimulates the mPTP opening and thereby induces mitochondrial dysfunctions in myocardial ischemia reperfusion [8]. The enzymatic activity of GSK-3*β* is regulated by phosphorylation. Phosphorylation at tyrosine 216 increases the activity, whereas phosphorylation at serine 9 significantly decreases the enzymatic activity of GSK-3*β* [7]. Moreover, phospho-GSK-3*β* (Ser9) suppresses pore formation via interacting with adenine nucleotide translocase (ANT), a major component of mPTP [9]. These results highlight the importance of mPTP dynamics in myocardial ischemia reperfusion injury.

**Figure 1:**
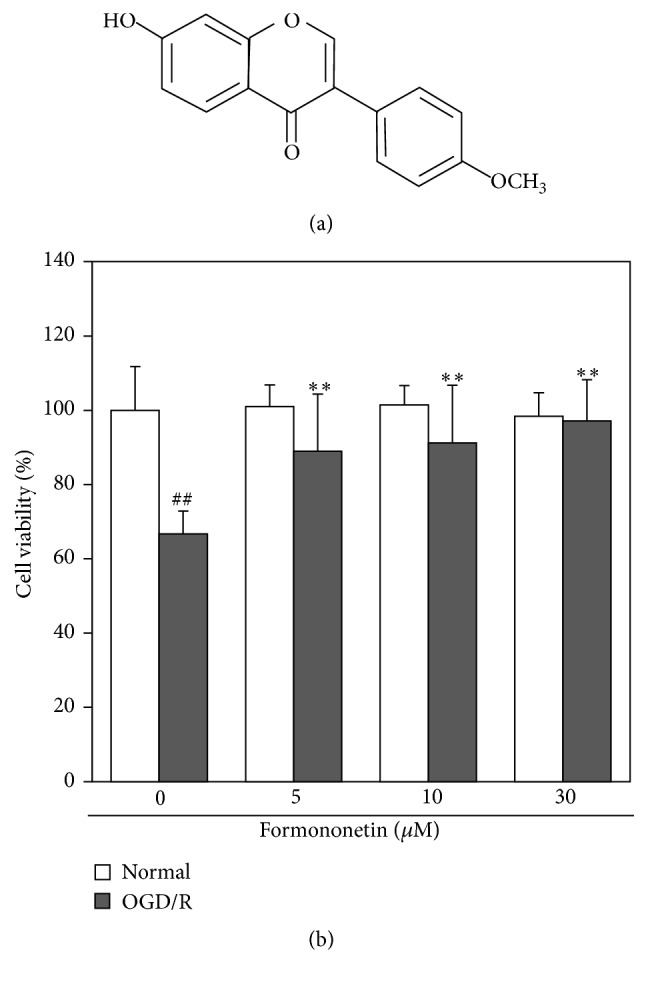
Formononetin protected cardiomyocyte H9c2 cells against OGD/reoxygenation injury. (a) Chemical structure of formononetin. (b) Effect of formononetin on the cell viability of H9c2 cells under normal or OGD/reoxygenation condition. H9c2 cells were treated with formononetin at various concentrations either under normal condition for 24 h (Normal) or subjected to OGD for 8 h followed by reoxygenation for 16 h (OGD/R). The cell viability was determined by MTT assay. The values represent mean ± SD (*n* = 5). ^##^ *p* < 0.01 (OGD/R versus Normal); ^*∗∗*^ *p* < 0.01 (drug versus OGD/R).

Medicinal plants such as* Radix Astragali* are widely used to treat cardiovascular diseases in traditional Chinese medicine [10, 11]. As a major isoflavone compound from* Radix Astragali*, formononetin bearing the structure shown in Figure 1(a) exhibits a wide range of pharmacological properties such as anticancer [12, 13], anti-inflammatory [14], antioxidant [15], antiviral [16], neuroprotective activities [17, 18], and wound healing [19]. However, little is known about the molecular mechanisms underlying the cardioprotective potential of formononetin within the context of myocardial infarction.

In the present study, we initially discovered that formononetin enhanced the survival of rat cardiomyocyte H9c2 cells during oxygen glucose deprivation (OGD) and reoxygenation. We hypothesize that formononetin may protect cardiomyocytes against ischemia reperfusion injury by preventing mPTP opening. We focused on the role of GSK-3*β* in the regulation of mPTP opening in H9c2 cells. We further examined the effects of formononetin on reactive oxygen species (ROS), PI3K/Akt, and PKC.

## 2. Materials and Methods

### 2.1. Materials and Regents

Formononetin was obtained from Yick-Vic Chemicals & Pharmaceuticals Ltd, (Hong Kong). Antibodies for GSK-3*β* and adenine nucleotide translocase (ANT) were purchased from Santa Cruz Biotechnology (Santa Cruz, CA, USA). Antibodies against phospho-GSK-3*β* (Ser9), glyceraldehydes-3-phosphate dehydrogenase (GAPDH), Akt, and phospho-Akt were obtained from Cell Signaling Technology (Boston, MA, USA). Anticyclophilin D (anti-Cyp-D) was purchased from Thermo Fisher Scientific Inc. (Waltham, MA, USA). Alexa Fluor 594-conjugated goat anti-rabbit IgG secondary antibody and Alexa Fluor 488-conjugated goat anti-mouse IgG secondary antibody were obtained from Invitrogen (Carlsbad, CA, USA).

### 2.2. Cell Culture

Rat cardiomyocyte H9c2 cell line was obtained from Shanghai Institute for Biological Sciences, Chinese Academy of Science (Shanghai, China), and cultured in Dulbecco's modified Eagle's medium (DMEM) supplemented with 10% heat-inactivated fetal bovine serum (Gibco/BRL, Gaithersburg, MD, USA), 2 mM glutamine, and 100 U/mL penicillin A/streptomycin at 37°C in a humidified incubator containing 5% CO_2_.

### 2.3. Measurement of ROS and Superoxide Production

H9c2 cells were seeded in 6-well plate at the density of 0.3 × 10^5^ cells/mL overnight. After 24 h incubation, the cells were exposed to OGD condition for 8 h and subsequently treated with formononetin at different concentrations under reoxygenation condition for 30 min. For the detection of intracellular ROS, the cells were stained with 5 *μ*M of 2′,7′-dichlorofluorescein-diacetate (DCFH2-DA) from Life Technologies (Grand Island, NY, USA) at 37°C for 30 min. On the other hand, the intracellular superoxide ion was detected with dihydroergotamine (DHE) from Invitrogen Molecular Probes (Eugene, OR, USA) at 37°C for 30 min. After the removal of excessive probes, the cells were imaged on a laser scanning fluorescence microscope (Carl-Zeiss, Jena, Germany). The results were expressed as a percentage of the intracellular ROS or superoxide production in OGD/reoxygenation-treated cells based on the analysis using NIH Image J software (http://imagej.nih.gov/ij/).

### 2.4. Imaging of Mitochondrial Membrane Integrity

Mitochondrial membrane integrity was detected by staining with tetramethylrhodamine methyl ester (TMRM) and calcein-AM as reported [20, 21]. Briefly, H9c2 cells were exposed to OGD condition for 8 h and treated with formononetin (5 to 30 *μ*M) under reoxygenation condition for another 20 min. H9c2 cells were incubated with 250 nM TMRM in medium for 15 min. For calcein-AM staining, the H9c2 cells were loaded with 1 *μ*M calcein-AM in the presences of 8 mM cobalt chloride for 30 min. After the removal of excessive dye by three washes with PBS, the cell monolayer was examined and imaged under a fluorescence microscope (Carl-Zeiss, Jena, Germany) through a 560 nm long-path filter.

### 2.5. Immunoprecipitation

Immunoprecipitation (IP) was performed as previously described [9]. In brief, 500 *μ*g of total cellular proteins from different treatment groups was incubated with 1 *μ*g primary antibodies against ANT and phospho-GSK-3*β* for 1 h at 4°C. The mixture was incubated with 20 *μ*L of protein A/G PLUS-agarose slurry (Santa Cruz, CA, USA) at 4°C overnight. The samples were subsequently centrifuged at 2500 rpm for 5 min at 4°C. The precipitations were recovered and washed with PBS buffer for 3 times. Finally, the pellets were dissolved in 60 *μ*L of 1x electrophoresis sample buffer and boiled for 5 min. Thirty microliters of each sample was analyzed by Western blotting.

### 2.6. Western Blot Analysis

The cellular proteins were extracted with ice-cold RIPA buffer according to the manufacturer's instructions (Sigma-Aldrich, St. Louis, MO, USA). In brief, 30 *μ*g of the cellular proteins was resolved in 10% SDS-PAGE and subsequently transferred onto polyvinylidene difluoride (PVDF) membrane (EMD Millipore, Billerica, MA, USA). Following the incubation in 5% BSA in TBST (50 mM Tris-Cl, 150 mM NaCl, 0.1% Tween-20, pH 7.6) at room temperature for 2 h, the membranes were probed with the primary antibodies against the indicated proteins (1 : 1,000 dilution) at 4°C overnight. After three washes with TBST buffer, the membranes were incubated with horseradish peroxidase- (HRP-) conjugated secondary antibody (1 : 1,000 dilution) at 4°C for 3 h and visualized by enhanced chemiluminescence (ECL) reaction reagents (GE Healthcare, Uppsala, Sweden).

### 2.7. Cell Immunofluorescence Imaging

H9c2 cells were seeded on a glass slide at the density of 0.5 × 10^4^ cells/slide. After 24 h incubation, the cells were exposed to OGD condition for 8 h and treated with 30 *μ*M formononetin under reoxygenation condition for another 20 minutes. At the end of treatment, H9c2 cells were fixed with 4% paraformaldehyde for 30 minutes, permeabilized with 0.5% Triton X-100 for 20 minutes, and blocked with 5% normal goat serum in PBS for 2 h at room temperature. Phospho-GSK-3*β* (Ser9) and mitochondrial membrane protein Tom20 were probed with specific primary antibodies overnight at 4°C. After 5 washes with PBS, the slides were incubated in the secondary antibodies (i.e., Alexa Fluor 594-conjugated goat anti-rabbit IgG secondary antibody and Alexa Fluor 488-conjugated goat anti-mouse IgG secondary antibody) for 90 min at room temperature. The cell nuclei were stained with DAPI. After the removal of excessive fluorescence reagents, the cells were imaged on a Zeiss fluorescence microscopy (Carl-Zeiss, Jena, Germany).

### 2.8. Statistical Analysis

The results were presented as means ± SD from three independent experiments. The data were compared by one-way analysis of variance (ANOVA), followed by least significant difference (LSD)* post hoc* test using IBM software SPSS version 20.0 (Amonk, NY, USA). Differences with *p* < 0.05 were considered as statistically significant.

## 3. Results

### 3.1. Formononetin Enhanced the Survival of H9c2 Cells against OGD/Reoxygenation Challenge

The effect of formononetin on the viability of H9c2 cells was measured by standard colorimetric MTT assay. Under normal conditions, formononetin at the concentration of 30 *μ*M did not affect cell viability over a period of 48 h. As shown in Figure 1(b), however, the cell viability was reduced to 66.7 ± 6.13% after 8 h of OGD and 16 h of reoxygenation. Interestingly, formononetin markedly increased the cell viability against OGD/reoxygenation-induced injury in a concentration-dependent manner.

### 3.2. Inhibitory Effect of Formononetin on ROS and Superoxide Production

**Figure 2:**
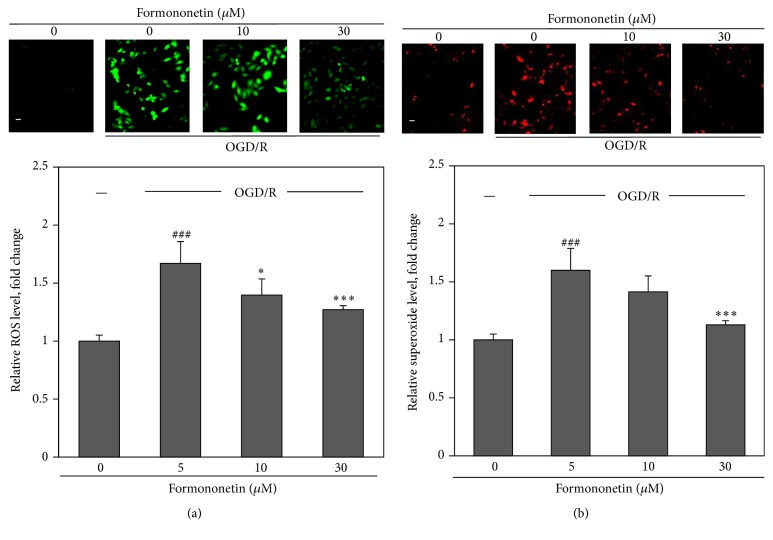
Effects of formononetin on ROS and superoxide production in cardiac H9c2 cells. H9c2 cells were treated with various concentrations of formononetin under OGD and reoxygenation (OGD/R) condition. Accumulation of ROS and superoxide in H9c2 cells was detected by probes DCFH2-DA and DHE, respectively. (a) Representative images and quantification of relative ROS levels. The intracellular ROS level in H9c2 cells was calculated based on fluorescence intensity. The results were expressed as means ± SD (*n* = 3). ^###^ *p* < 0.001 (OGD/R versus Normal); ^*∗*^ *p* < 0.05; ^*∗∗∗*^ *p* < 0.001 (drug versus OGD/R). Scale bar, 10 *μ*m. (b) Representative images and quantification of relative superoxide levels. The intracellular superoxide level in H9c2 cells was calculated based on fluorescence intensity. The fluorescence images were analyzed with NIH Image J software (http://imagej.nih.gov/ij/). The results were expressed as means ± SD (*n* = 3). ^###^ *p* < 0.001 (OGD/R versus Normal); ^*∗*^ *p* < 0.05; ^*∗∗∗*^ *p* < 0.001 (drug versus OGD/R). Scale bar, 10 *μ*m.

The effect of formononetin on the intracellular redox status was determined by detecting the intracellular H_2_O_2_ and superoxide ion. Upon the introduction into the cells, the nonpolar probe DCFH2-DA was converted into the polar intermediate DCFH by cellular esterases and further oxidized to highly fluorescent product 2′,7′-dichlorofluorescein (DCF) by the intracellular H_2_O_2_ and other types of ROS. On the other hand, superoxide anion reacts with DHE, generating a red fluorescent product 2-hydroxyethidium (EOH). As shown in Figures 2(a) and 2(b), OGD/reoxygenation dramatically increased the generation of H_2_O_2_ and superoxide ion in H9c2 cells. Based on the fluorescence intensity, we estimated that OGD/reoxygenation increased H_2_O_2_ level by 1.7-fold and also enhanced superoxide ion formation by 1.6-fold relative to the untreated controls. However, formononetin attenuated the effects of OGD/reoxygenation on the generation of H_2_O_2_ and superoxide ion in a concentration-dependent manner. Formononetin at 30 *μ*M reduced OGD/reoxygenation-induced production of H_2_O_2_ and superoxide ion down to 1.3-fold and 1.2-fold, respectively.

### 3.3. Effect of Formononetin on Mitochondrial Membrane Integrity

**Figure 3:**
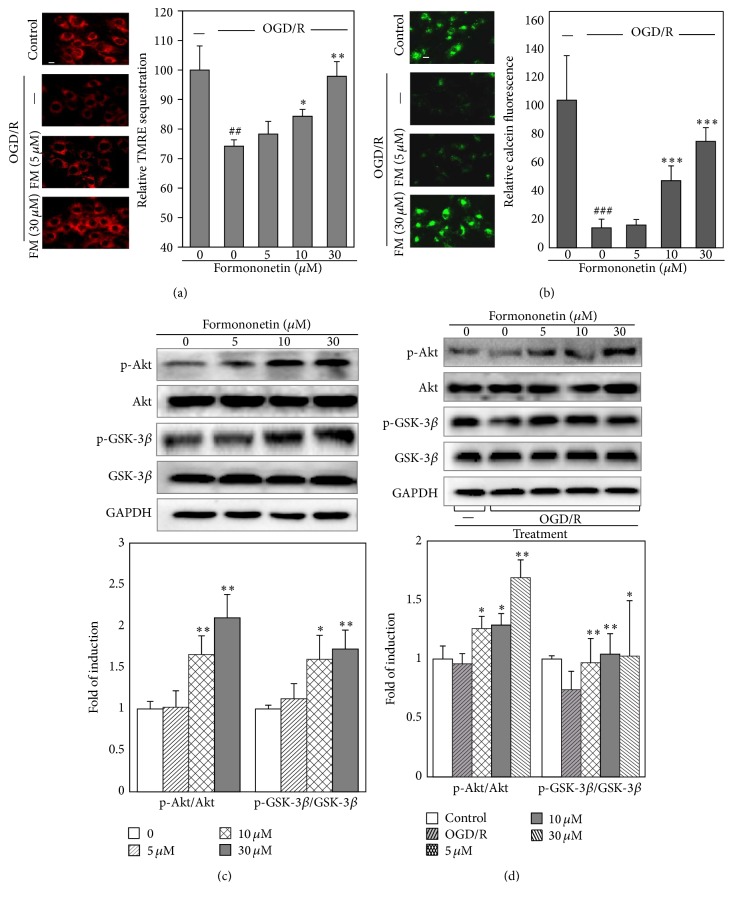
Effects of formononetin on mitochondrial membrane integrity and phosphorylation of Akt and GSK-3*β*. (a) Fluorescence images and quantification of TMRM sequestration in mitochondria. H9c2 cells were treated with formononetin (5, 10, and 30 *μ*M) under normal or OGD and reoxygenation condition and stained with TMRM. TMRM fluorescence intensity was determined by NIH image J software. Results are presented as means ± SD (*n* = 3). ^##^ *p* < 0.01 (OGD/R versus normal); ^*∗*^ *p* < 0.05, ^*∗∗*^ *p* < 0.01 (drug versus OGD/R). Scale bar, 10 *μ*m. (b) Fluorescence images and quantification of calcein sequestration in mitochondria. H9c2 cells were treated similarly as described in (a) and stained with calcein-AM. Calcein fluorescence intensity was determined by NIH image J software. Results are presented as means ± SD (*n* = 3). ^###^ *p* < 0.001 (OGD/R versus normal); ^*∗∗∗*^ *p* < 0.001 (drug versus OGD/R). Scale bar, 10 *μ*m. (c) Western blot analysis and quantification of phosphorylation of GSK-3*β* and Akt in response to formononetin. After the treatment with formononetin (5, 10, and 30 *μ*M) under normal condition, H9c2 cells were lysed and subsequently analyzed for phosphorylation of GSK-3*β* (Ser9) and Akt by Western blotting using specific antibodies. Western blots were quantified by a densitometric method. Results are presented as means ± SD (*n* = 3). ^*∗*^ *p* < 0.05, ^*∗∗*^ *p* < 0.01 (drug versus control). (d) Western blot analysis and quantification of phosphorylation of GSK-3*β* and Akt in response to formononetin under normal or OGD/R condition. After the treatment with formononetin (5, 10, and 30 *μ*M) under normal or OGD/R condition, H9c2 cells were lysed and subsequently analyzed for phosphorylation of GSK-3*β* (Ser9) and Akt by Western blotting using specific antibodies. Western blots were quantified by a densitometric method. Results are presented as means ± SD (*n* = 3). ^*∗*^ *p* < 0.05; ^*∗∗*^ *p* < 0.01 (drugs versus control and drugs versus OGD/R).

To determine the effect of formononetin on mitochondrial membrane integrity, a cell-permeant and cationic dye TMRM was used to localize active mitochondria. As shown in Figure 3(a), after exposure to OGD for 8 h and reoxygenation for 20 min, the capability of mitochondria to sequester TMRM was markedly diminished to 74.2 ± 2.2% relative to untreated control cells. Formononetin preserved the sequestration of TMRM by active mitochondria in a concentration-dependent manner. After the treatment with 30 *μ*M formononetin, H9c2 cells showed the recovery of TMRM fluorescence to 97.9 ± 5.0% relative to untreated control cells. Calcein-AM staining showed similar results as shown in Figure 3(b).

### 3.4. Effects of Formononetin on Akt and GSK-3*β* Phosphorylation

To discover the potential mechanisms, we first examined the effects of formononetin on the phosphorylation of Akt and GSK-3*β*, especially the phosphorylation of GSK-3*β* at Ser9. As shown in Figure 3(c), formononetin at the concentrations (5–30 *μ*M) induced the phosphorylation of Akt and GSK-3*β* at Ser9 in a concentration-dependent manner. We further verified the effects of formononetin on the phosphorylation of Akt and GSK-3*β* at Ser9 in the cells after exposure to OGD and reoxygenation condition. As shown in Figure 3(d), the levels of phospho-Akt and phospho-GSK-3*β* (Ser9) were suppressed under OGD/reoxygenation condition. However, formononetin increased Akt phosphorylation against OGD/reoxygenation stimulation in a concentration-dependent manner. Interestingly, formononetin even at the concentration of 5 *μ*M effectively reversed the loss of GSK-3*β* phosphorylation against OGD/reoxygenation challenge.

### 3.5. Formononetin Activated Akt and PKC to Preserve Mitochondrial Integrity

**Figure 4:**
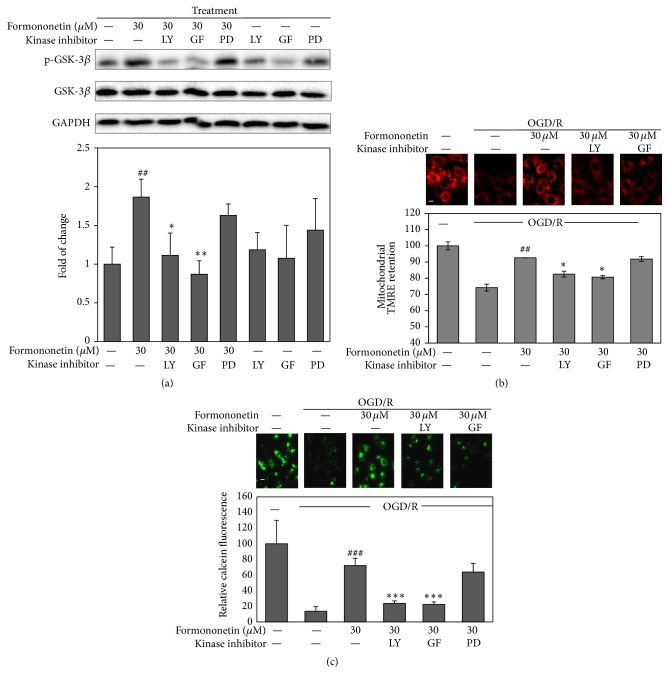
Roles of PI3K, ERK1/2, and PKC in the regulation of GSK-3*β* phosphorylation and mitochondrial membrane integrity by formononetin. (a) Western blot analysis and quantification of GSK-3*β* phosphorylation. After the treatment with formononetin ± kinase inhibitors, H9c2 cells were lysed and analyzed for GSK-3*β* phosphorylation at Ser9 by Western blotting using specific antibodies. Western blots were determined by a densitometric method. Results are presented as means ± SD (*n* = 3). ^##^ *p* < 0.01 (drug versus OGD/R); ^*∗*^ *p* < 0.05, ^*∗∗*^ *p* < 0.01 (drug + inhibitor versus drug). (b and c) Fluorescence images and quantification of TMRM and calcein sequestration in mitochondria. After the treatment with formononetin ± kinase inhibitors under OGD and reoxygenation condition, H9c2 cells were stained with TMRM and calcein-AM and imaged under a fluorescence microscope (Carl-Zeiss, Jena, Germany). TMRM and calcein fluorescence intensity were quantified by a densitometric method. ^##^ *p* < 0.001 (drug versus OGD/R), ^*∗*^ *p* < 0.05 (drug + inhibitor versus drug). LY, PI3K inhibitor LY294002; GF, PKC inhibitor GF109203X; PD, ERK 1/2 inhibitor PD98059. Scale bar, 10 *μ*m.

To understand how formononetin induces the phosphorylation of GSK-3*β* at Ser9, following OGD/reoxygenation challenge, we treated the cells with formononetin in combination with PI3K inhibitor LY294002 (LY), PKC inhibitor GF109203X (GF), and ERK 1/2 inhibitor PD98059 (PD). As shown in Figure 4(a), PI3K inhibitor LY and PKC inhibitor GF almost abolished the stimulatory effect of formononetin on the phosphorylation of GSK-3*β* at Ser9, whereas ERK1/2 inhibitor did not show any activity against formononetin-induced formation of phospho-GSK-3*β* (Ser9). Supportively, PI3K inhibitor LY and PKC inhibitor GF effectively antagonized the effects of formononetin on mitochondrial retention of TMRM and calcein-AM against OGD/reoxygenation damage (Figures 4(b) and 4(c)).

### 3.6. Formononetin Stimulated the Binding of p-GSK-3*β* to ANT in mPTP

**Figure 5:**
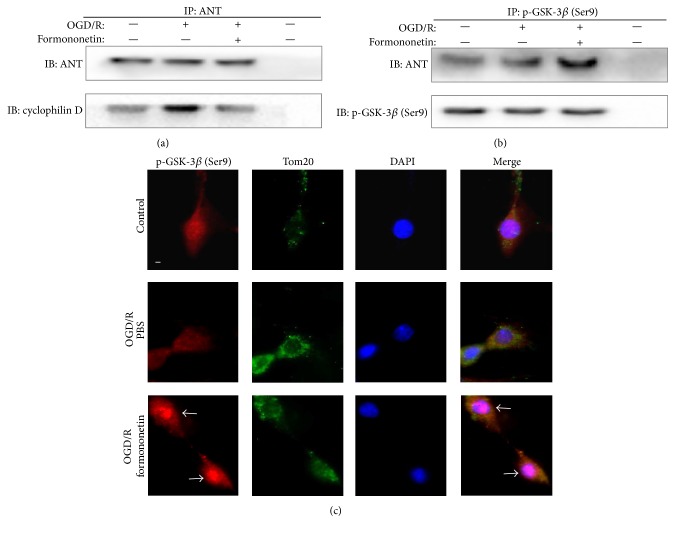
Effects of formononetin on the formation of ANT-Cyp-D complex and the binding of phospho-GSK-3*β* (Ser9) to ANT. (a) Immunoprecipitation with antibody against ANT. After the treatment with formononetin under OGD and reoxygenation condition, H9c2 cells were lysed for immunoprecipitation (IP) with antibody against ANT. The pull-down materials were analyzed by immunoblotting (IB) with antibodies against ANT and Cyp-D. (b) Immunoprecipitation with antibody against phospho-GSK-3*β* (Ser9). After the treatment with formononetin under OGD and reoxygenation condition, H9c2 cells were lysed for immunoprecipitation (IP) with antibody against phospho-GSK-3*β* (Ser9). The pull-down materials were analyzed by immunoblotting (IB) with antibodies against ANT and phospho-GSK-3*β* (Ser9). (c) Immunofluorescence imaging of the intracellular phospho-GSK-3*β* (Ser9). After the treatment with formononetin under OGD and reoxygenation condition, H9c2 cells were probed with antibody against phospho-GSK-3*β* (Ser9) and mitochondrial membrane protein Tom20 and subsequently detected by fluorophore-labelled secondary antibodies, phospho-GSK-3*β* (Ser9) (RED), and Tom20 (GREEN), respectively. The cell nuclei were indicated by DAPI staining. The images were captured under a fluorescence microscope (Carl-Zeiss, Jena, Germany). Scale bar, 10 *μ*m.

To verify the effect of formononetin on the mPTP opening, we examined the formation of protein complex between two main mPTP components ANT and Cyp-D. Following immunoprecipitation with anti-ANT antibody, we analyzed the presence of ANT and Cyp-D by Western blotting with specific antibodies. As shown in Figure 5(a), OGD/reoxygenation increased the formation of ANT-Cyp-D complex, whereas formononetin diminished OGD/reoxygenation-induced formation of ANT-Cyp-D complex. On the other hand, we performed immunoprecipitation with anti-phospho-GSK-3*β* (Ser9) antibody and subsequently analyzed the presence of ANT and phospho-GSK-3*β* (Ser9) by Western blotting with specific antibodies. We found that formononetin markedly increased the binding of phospho-GSK-3*β* (Ser9) to ANT (Figure 5(b)).

The binding of phospho-GSK-3*β* (Ser9) to mitochondrial membrane was further characterized by immunofluorescence staining. After 8 h of OGD, H9c2 cells were treated with 30 *μ*M formononetin or equal volume of PBS under reoxygenation condition for 20 min. The intracellular phospho-GSK-3*β* (Ser9) was sequentially stained by specific antibody and Alexa Fluor 594-conjugated goat anti-rabbit IgG secondary antibody. Mitochondria were indicated by sequential staining of Tom20 with anti-Tom20 and Alexa Fluor 488-conjugated goat anti-mouse IgG secondary antibody. DAPI was used to visualize the cell nuclei. As shown in Figure 5(c), formononetin dramatically increased the localization of phospho-GSK-3*β* (Ser9) in mitochondria in close proximity to the cell nucleus.

## 4. Discussion

Reperfusion of ischemic hearts is the main cause of the opening of mPTP, leading to the dysfunction of mitochondria [22]. The present study investigated that the effects of plant natural product formononetin on the mPTP opening in an* in vitro* OGD/reoxygenation-induced cell model of ischemia reperfusion injury. Following OGD challenge, we mimicked reperfusion by incubating the cells with drugs under reoxygenation condition as previously described [23]. We discovered that formononetin could enhance the survival of H9c2 cells and inhibit the mPTP opening through the potential mechanisms involving the inhibition of ROS production and Akt- and PKC-mediated phosphorylation of GSK-3*β* at Ser9.

Formononetin, a major isoflavone from* Radix Astragali*, was recently found to significantly reduce the infracted volume and the brain water accumulation in a rat model of ischemia-reperfusion injury via the activation of PI3K/Akt signaling pathway [17]. Sulphonated formononetin also showed cardioprotective effect on acute myocardial infarction in rats possibly by regulating energy metabolism in cardiac mitochondria [24]. Inhibition of mPTP opening was recently suggested as a potential therapeutic target for the development of new cardioprotective drugs against myocardial infarction [25, 26]. These results stimulated us to explore whether formononetin could exhibit cardioprotective effects by regulating mPTP opening. In this study, we focused on the effects of formononetin on OGD/Reoxygenation-induced disruption of mitochondrial membrane integrity and the underlying mechanisms. We first verified that formononetin could effectively suppress the overproduction of ROS and superoxide ion after OGD/reoxygenation challenge. We characterized the effects of formononetin on the phosphorylation of GSK-3*β* (Ser9). Phosphorylation of GSK-3*β* at Ser9 causes the reduction of GSK-3*β* activity [27]. Inactivation of GSK-3*β* is known to modulate the mPTP opening, conferring cardioprotection during ischemic preconditioning and postconditioning [9, 28].

In the present study, we found that formononetin markedly induced the phosphorylation of GSK-3*β* at Ser9 in a concentration-dependent manner. Presumably, formononetin could inactivate the enzymatic role of GSK-3*β* in cardiac H9c2 cells. Importantly, formononetin could recover the loss of phospho-GSK-3*β* (Ser9) formation against OGD/Reoxygenation challenge. To elucidate the mechanisms by which formononetin stimulates the formation of phospho-GSK-3*β* (Ser9), we explored the roles of the PI3K/Akt and PKC pathways. It was previously demonstrated that PI3K/Akt and PKC directly phosphorylated GSK-3*β* at Ser9 [29]. Another recent study reported that formononetin protected cerebra against ischemia/reperfusion injury via the activation of PI3K/Akt signaling pathway [17]. Indeed, formononetin induced the activation of Akt in a concentration-dependent manner. Formononetin at the concentration of 30 *μ*M achieved the most effective induction of Akt activation. On the other hand, formononetin at the concentration of 5 *μ*M already strongly promoted the formation of of phospho-GSK-3*β* (Ser9). Thus, we employed specific kinase inhibitors to profile the actions of Akt, ERK1/2, and PKC on the phosphorylation of GSK-3*β* at Ser9. Consequently, we discovered that both Akt and PKC contributed to the phosphorylation of GSK-3*β* at Ser9. Importantly, PI3K inhibitor LY294002 and PKC inhibitor GF109203X not only antagonized the stimulatory effects of formononetin on the phosphorylation of GSK-3*β* at Ser9 but also attenuated the protective effects of formononetin on mitochondrial membrane integrity. These results highlight that formononetin may maintain mitochondrial membrane integrity via inducing the phosphorylation of GSK-3*β* at Ser9 through Akt- and PKC-dependent mechanisms. We further demonstrated that formononetin induced the disassociation of ANT-Cyp-D complex and promoted the binding of phospho-GSK-3*β* (Ser9) to ANT. Moreover, GSK-3*β* is a multifunctional enzyme that regulates several cellular functions and transcription factors [30]. Nuclear GSK-3*β* level is markedly increased prior to the activation of the caspase cascade leading to apoptosis [31]. Interestingly, our immunofluorescence analysis suggested that phospho-GSK-3*β* (Ser9) might be deposited in mitochondria in close proximity to the cell nucleus. The deposition of p-GSK-3*β* at the interface between mitochondria and cell nucleus may limit the flux of ROS into cell nucleus and thereby suppress cell apoptosis.

The mPTP opening may be regulated by posttranslocational modification of Cyp-D and physical interaction of Cyp-D with matrix proteins (e.g., p53, PPAR alpha) [32]. It was recently reported that active GSK-3*β* could phosphorylate Cyp-D and stimulate the opening of mPTP [33]. Conversely, the suppression of GSK-3*β* resulted in the inhibition of mPTP opening by attenuating the phosphorylation of Cyp-D in murine tubular epithelial cells [34]. In the present study, we found that formononetin could induce GSK-3*β* phosphorylation at Ser9, causing the loss of GSK-3*β* enzyme activity. Taken together, it is possible that formononetin inhibited the phosphorylation of Cyp-D, contributing to the inhibition of mPTP opening. In addition to ANT, Cyp-D interacts with several other proteins (e.g., p53) to induce PTP opening and necrosis. Tumor suppressor protein p53 is accumulated in the mitochondrial matrix and interacts with Cyp-D during ischemia/reperfusion injury. Conversely, the reduction of p53 is protective during stroke due to the loss of Cyp-D and p53 complex [35]. Oxidative stress could induce the formation of Cyp-D-PPAR*α* complex in cardiomyocytes, while the disruption of Cyp-D-PPAR*α* complex by metformin could protect cardiomyocytes against oxidative stress-induced cell death [36]. Moreover, recent studies provided new evidences to support the roles of Cyp-D and F1F0-ATPase in the control of mPTP opening. F1F0-ATPase mainly affects mitochondrial ATP storage [37]. Suppression of the F1F0-ATPase in the mitochondrial matrix could conserve energy during the cardioprotection intervention of Bcl-2 or GSK-3*β* inhibitors, although GSK-3*β* inhibitors could not affect the activity of F1F0-ATPase [38]. Others also demonstrated a central role for mitochondrial GSK-3*β* but not F1F0-ATPase as target of PKC*ξ* to limit I/R damage [39]. In the present study, we discovered that formononetin could disrupt the interaction between ANT and Cyp-D. The disassociation of ANT from Cyp-D likely indicates that formononetin dismantles the mPTP complex via stimulating GSK-3*β* phosphorylation at Ser9 and inhibiting the enzymatic activity of GSK-3*β*. Further work is required to clarify (1) whether formononetin affects the interactions of Cyp-D with p53 and PPAR*α* and (2) whether formononetin affects the activity of mitochondrial F1F0-ATPase.

In addition, it was reported that the phosphorylation of GSK-3*β* at Ser9 suppressed mPTP opening via interacting with and phosphorylating VDAC [40]. Activated GSK-3*β* also phosphorylates VDAC and thereby disrupts the binding of hexokinase II to mitochondrial components [41]. However, the exact role of VDAC in the regulation of mPTP opening remains elusive. The results from our study defined that formononetin inhibited the activity of GSK-3*β* by Ser9 phosphorylation and did not exclude the interactions of GSK-3*β* and phospho-GSK-3*β* (Ser9) with VDAC.

**Figure 6:**
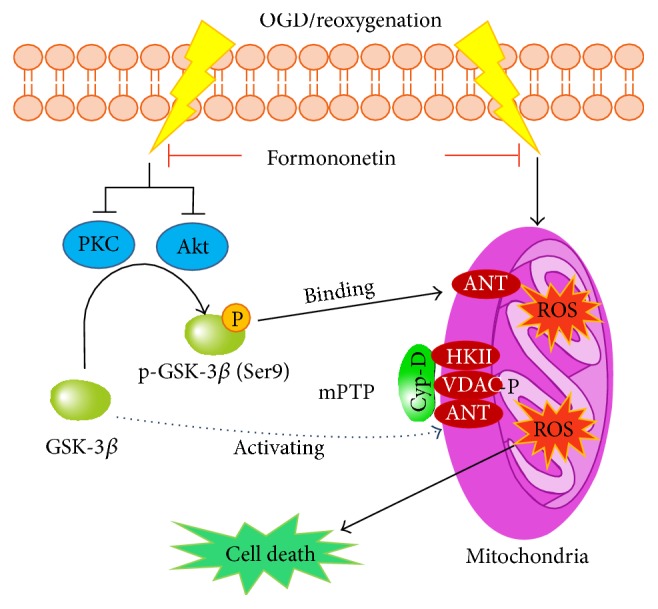
Proposed mechanism underlying the cardioprotective effect of formononetin. Formononetin activates PI3K/Akt and PKC signaling pathways and subsequently induces the phosphorylation of GSK-3*β* at Ser9. The phosphorylation of GSK-3*β* at Ser9 leads to the inactivation of GSK-3*β* in the opening of mPTP. On the other hand, phospho-GSK-3*β* (Ser9) migrates towards mitochondria and binds to ANT, causing the disassociation of the mPTP complex involving ANT, Cyp-D, VDAC, and HKII. Formononetin also inhibits the overproduction of ROS and superoxide, possibly reducing the oxidative damage of mitochondria in cardiomyocytes.

In summary, the results from the present study suggest that formononetin may protect cardiomyocytes against ischemia reperfusion injury by inhibiting the mPTP opening. The potential underlying mechanisms could be outlined in Figure 6. Formononetin may initiate its actions by suppressing the production of ROS and superoxide ion and activating Akt and PKC pathways. Upon activation, Akt and PKC subsequently phosphorylate GSK-3*β* at Ser9, leading to direct inhibition of GSK-3*β* activity towards the mPTP opening and the disruption of the protein complex involving ANT and Cyp-D. Ultimately, formononetin attenuated the mPTP opening and maintained mitochondrial membrane integrity. Thus, formononetin may serve as a useful lead compound for future development of new cardioprotective drugs against acute myocardial infarction.
